# Association of handgrip strength weakness and asymmetry with cognitive impairment and depressive symptoms in older Chinese adults

**DOI:** 10.1038/s41598-025-93573-6

**Published:** 2025-03-21

**Authors:** Nazhakaiti Abudukelimu, Peng Zhang, Jing Du, Min Li, Yupei Shen, Yanyan Mao, Difei Wang, Qianxi Zhu

**Affiliations:** 1 Shanghai Institute for Biomedical and Pharmaceutical Technologies, School of Public Health, Fudan University, Shanghai, 200237 China; 2 Shanghai-MOST Key Laboratory of Health and Disease Genomics, NHC Key Lab of Reproduction Regulation, Shanghai Institute for Biomedical and Pharmaceutical Technologies, Shanghai, 200237 China; 3 School of Management, Hainan Medical University, Haikou, 571199 China; 4 School of Clinical Medicine, Shanghai University of Medicine and Health Sciences, Shanghai, 201318 China

**Keywords:** Older adults, Cognitive impairment, Depression, Handgrip strength, Mental status and dementia tests, Diseases, Medical research

## Abstract

This study investigated the association between handgrip strength (HGS) asymmetry and weakness with cognitive function and depressive symptoms among 920 community-dwelling adults aged above 60 years in suburban Shanghai. Participants were selected using a multistage cluster-stratified sampling approach. Assessments included HGS measured with a dynamometer, the Montreal Cognitive Assessment (MoCA) for cognition, and the Geriatric Depression Scale (GDS) for depressive symptoms. Restricted cubic splines revealed a positive association between dominant HGS and MoCA scores, indicating better cognitive performance, and a negative association with GDS scores, suggesting fewer depressive symptoms. The association between the HGS ratio and MoCA scores and the HGS ratio and GDS scores varied by sex. Women with HGS weakness alone (odds ratio (OR) = 2.00, 95% confidence interval (CI) = 1.17–3.37), asymmetry alone (OR = 1.93, 95% CI = 1.14–3.29), or weakness and asymmetry together (OR = 2.57, 95% CI = 1.48–4.46) had a significantly increased risk of cognitive impairment. However, no such associations observed in men. These findings suggest that HGS weakness and asymmetrical HGS may be associated with a higher risk of cognitive decline and depressive symptoms, particularly in women. This study emphasizes the need for sex-specific assessments and prevention strategies to address cognitive and mental health issues among older adults.

## Introduction

The aging of the global population amplifies the significance of health issues among older adults, including cognitive decline, depression, frailty, coronary heart disease, hypertension, diabetes, sarcopenia, multimorbidity, and other conditions^1–5^. Studies have shown that the prevalence of mild cognitive impairment (MCI) and depressive symptoms among older adults in China has reached 15.5%^6^and 20.0%^7^, respectively. Cognitive disorders, such as Alzheimer’s disease and MCI, as well as affective disorders like depression, significantly impact older adults’ self-sufficiency and social engagement^8,9^. The substantial burden these conditions place on families and healthcare systems underscores their importance as key areas of public health research.

Recent research highlights the close association between cognitive disorders, depression, and physiological decline in older adults, including reduced muscle mass, decreased muscle strength, and slower gait^10–12^. Although gait speed and muscle mass assessments are valuable, they may be limited by individual lower limb mobility and healthcare facility capabilities^13,14^. Handgrip strength (HGS) offers a stable, convenient, and cost-effective method for assessing muscle strength and has become a focus of increasing research^15^.

Despite the growing interest in the association between HGS and cognitive and depressive status, a notable concern is the variety of HGS measurement methodologies across studies, particularly the neglect of asymmetry between the dominant and non-dominant hands^16^. Measuring HGS asymmetry provides critical information for a more refined understanding of muscle strength and asymmetry. This study aimed to address these gaps and explore the associations between dominant HGS, HGS asymmetry, and cognitive and depressive status.

## Methods

### Study population

This study, using a multistage cluster-stratified sampling method, focused on community-dwelling older adults aged 60–90 years living in suburban Shanghai with no history of psychotic disorders or limited mobility in 2019. The specific sampling method can be found in the previous study^17^. The study was approved by the Ethics Committee of the Shanghai University of Medicine & Health Sciences (2019-SMHC-01–003) and was conducted strictly in accordance with the Declaration of Helsinki. All research activities adhered to the ethical standards, including obtaining written informed consent from participants prior to data collection.

## Measurements

HGS assessment.

HGS was assessed using a mechanical dynamometer as part of a comprehensive medical examination. Participants were instructed to position their elbows at a 90-degree angle and apply maximal force to the dynamometer for several seconds, alternating between both hands. The dominant and non-dominant hands were identified based on comparative HGS measurements, with the hand exhibiting the greatest force designated as the dominant hand. According to the 2019 Asian Sarcopenia Consensus, HGS weakness is classified as an HGS of less than 28 kg in men and less than 18 kg in women^18^. The HGS ratio was calculated by dividing the dominant HGS by the non-dominant HGS (dominant HGS, kg/non-dominant HGS, kg). Participants exhibiting a ratio of 1.00–1.10 were characterized as having HGS symmetry, whereas those with a ratio > 1.10 were identified as having HGS asymmetry. The participants were classified into four HGS status groups: neither weakness nor asymmetry, weakness alone, asymmetry alone, and weakness and asymmetry together.

Clinical assessment.

Cognitive function was evaluated using the Montreal Cognitive Assessment (MoCA) with a 30-point scale^19,20^. This scale effectively categorizes our study population into two distinct groups based on scores. The cut-off scores varied depending on education level: 13 for illiterate, 19 for primary school graduates, and 24 for middle school or higher^21^. Individuals scoring above these cut-off values are considered to have normal cognitive function, while those scoring below are classified as having cognitive impairment. The Geriatric Depression Scale (GDS) comprises 30 items, with scores ranging from 0 to 30; scores 10 or above are categorized as indicative of depression, while below 10 are considered normal^22^. The Mini Nutritional Assessment (MNA) includes general, anthropometric, dietary, and subjective assessments^23^. The scale was calibrated using reference values ranging from 0 to 30. All the assessment scales mentioned above are Chinese versions.

Covariates assessment.

The following socio-demographic covariates were included: sex, age, education level (“illiteracy,” “primary school,” “junior high school or higher.”), pre-retirement occupation (“non-manual,” “manual,” and “mixed.”), living arrangement (“living alone,” and “not living alone.”), and income (“less than ¥1000 per month,” “¥1000–¥3000 per month,” “¥3000–¥5000 per month,” and “more than ¥5000 per month.”). These factors were assessed through face-to-face interviews using standardized questionnaires. Data on biological characteristics such as body mass index (BMI) were collected during medical checkups.

### Statistical analysis

Continuous variables are presented as means with standard deviations or medians with interquartile ranges (IQR), and categorical variables are expressed as percentages. Analysis of variance (ANOVA) and chi-square tests were used to evaluate differences in baseline sociodemographic, lifestyle, and health characteristics.

Linear regression models with restricted cubic splines (RCS) were fitted to assess nonlinearity among dominant HGS, HGS ratio, MoCA score, and GDS score. The optimal number of knots was determined based on the Akaike Information Criterion, Bayesian Information Criterion, and changes in the R² values of the model. It was found that the RCS with three knots was adequate for capturing nonlinear patterns in the data without overfitting.

Multivariate logistic regression analyses were conducted to calculate the odds ratios (ORs) and their corresponding 95% confidence intervals (CIs) for the association between the HGS status and cognitive impairment and depression. The covariates adjusted in the RCS and multivariate logistic regression models included age, BMI, living arrangements, education level, pre-retirement occupation, MNA score, and income. All statistical analyses were performed using SAS statistical software (version 9.4; SAS Institute Inc., Cary, NC, USA) and R (version 4.3.3, https://www.R-project.org/). Tests were two-tailed, with a significance level of *P* < 0.05.

## Results

After excluding individuals with incomplete data, a total of 920 individuals aged over 60 years were included in our study (men: 42.3%; women: 57.7%). The participants were divided into the following age groups: 60–69 years (370 participants), 70–79 years (450 participants), and 80–89 years (100 participants). The range of age, MoCA, GDS, dominant HGS, non-dominant HGS, and HGS ratio were 60–89 years, 2–30, 0–29, 5.10–44.90 kg, 4.80–43.40 kg, 1.00–1.30, respectively. The median (IQR) age, MoCA, GDS, dominant HGS, non-dominant HGS, and HGS ratio were 71 (66–75) years, 19 (15–23), 5 (2–8), 22.65 (17.73–29.48) kg, 21.00 (16.10–27.60) kg, and 1.07 (1.03–1.12), respectively.

ANOVA demonstrated that MoCA scores, dominant HGS, and non-dominant HGS declined with increasing age (all *P* < 0.001), lower education levels (all *P* < 0.001), and in individuals living alone (all *P* < 0.01). GDS scores were higher in lower-income groups (*P* = 0.004) and individuals living alone (*P* < 0.001). The HGS ratio was higher in groups with lower education level (*P* < 0.001) and those living alone (*P* = 0.010). Sex was a significant factor influencing the results of all five assessments (all *P* < 0.01), with women showing adverse outcomes (Table 1).

**Table 1:** Comprehensive statistical distribution of MoCA, GDS, and HGS indicators in older adults.

	*n* (%)	Age		MoCA		GDS		Dominant HGS		Non-Dominant HGS		HGS Ratio	
		Mean	*P*	Mean	*P*	Mean	*P*	Mean	*P*	Mean	*P*	Mean	*P*
All		71.92		18.29		5.77		23.53		21.84		1.09	
Age group					< 0.001		0.063		< 0.001		< 0.001		0.073
60–69	370 (40.2)	/		19.82	a	5.85		24.84	a	21.01	a	1.09	
70–79	450 (48.9)	/		18.23	b	5.50		23.54	b	19.29	b	1.08	
80–89	100 (10.9)	/		12.92	c	6.69		18.71	c	17.15	c	1.10	
Sex			0.717		< 0.001		0.008		< 0.001		< 0.001		< 0.001
Men	389 (42.3)	72.00		19.89		5.29		29.97		28.03		1.07	
Women	531 (57.7)	71.87		17.12		6.12		18.82		17.31		1.09	
Education			< 0.001		< 0.001		0.167		< 0.001		< 0.001		< 0.001
Illiteracy	345 (37.5)	73.23	a	13.90	a	6.02		21.03	a	19.33	a	1.10	a
Primary school	317 (34.5)	71.00	b	19.84	b	5.85		23.83	b	22.10	b	1.08	b
Junior high school or higher	258 (28.0)	71.31	c	22.27	c	5.31		26.53	c	24.88	c	1.07	c
Pre-retirement occupation			0.876		< 0.001		0.944		< 0.001		< 0.001		0.038
Non-manual	79 (8.6)	72.00		22.01	a	5.59		28.26	a	26.61	a	1.07	a
Manual	764 (83.0)	71.89		17.55	b	5.78		22.74	b	21.08	b	1.09	b
Mixed	77 (8.4)	72.22		21.74	c	5.74		26.43	a	24.43	a	1.09	ab
Income (¥/month)			0.074		< 0.001		0.004		0.001		< 0.001		0.096
< 1000	85 (9.2)	73.16		15.34	a	7.09	a	21.81	a	20.17	a	1.09	
1000 ~ 3000	541 (58.8)	71.59		17.80	b	5.90	b	23.06	a	21.35	a	1.09	
3000 ~ 4000	135 (14.7)	72.21		19.18	c	5.40	bc	23.97	ab	22.20	a	1.08	
> 5000	159 (17.3)	72.13		20.84	d	4.94	cd	25.68	b	24.09	b	1.07	
Living arrangement			< 0.001		< 0.001		< 0.001		< 0.001		< 0.001		0.010
Not living alone	763 (82.9)	71.42		18.71		5.45		24.14		22.45		1.08	
Living alone	157 (17.1)	74.34		16.27		7.28		20.59		18.86		1.10	

MoCA: the Montreal Cognitive Assessment; GDS: the Geriatric Depression Scale; Dominant HGS: dominant handgrip strength; Non-Dominant HGS: non-dominant handgrip strength; HGS Ratio: handgrip strength ratio (dominant HGS, kg/non-dominant HGS, kg).

*If the ANOVA indicated a significant difference, then the Least Significant Difference (LSD) method was employed to conduct post-hoc multiple-comparisons analysis. Significant differences between pairs of groups are denoted by different letters (a, b, c and d).

**Fig. 1:**
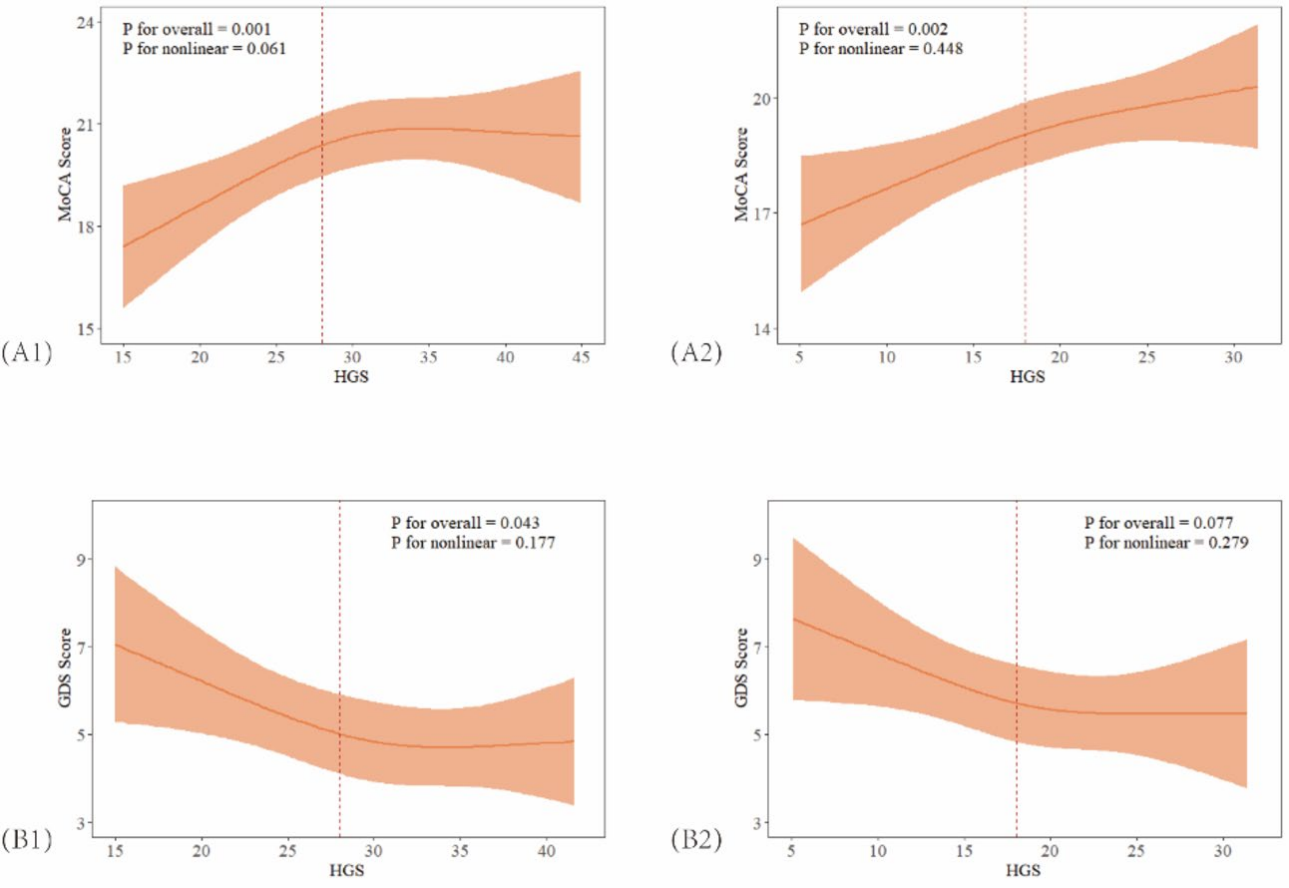
Associations of Dominant HGS with MoCA And GDS Scores. (A1) Association of Dominant HGS with MoCA Scores in Men. (A2) Association of Dominant HGS with MoCA Scores in Women. (B1) Association of Dominant HGS with GDS Scores in Men. (B2) Association of Dominant HGS with GDS Scores in Women. All adjusted for age, BMI, living arrangement, education levels, pre-retirement occupation, MNA scores, income. HGS: handgrip strength; MoCA: the Montreal Cognitive Assessment; GDS: the Geriatric Depression Scale; BMI: Body mass index; MNA: the Mini Nutritional Assessment.

RCS analysis demonstrated positive associations between dominant HGS and MoCA scores in both men and women, with the association plateauing at 32 kg in men (Fig. 1A1 and 1A2). A negative association between dominant HGS and GDS scores was observed in both groups, with the association flattening after 30 kg in men and 20 kg in women (Fig. 1B1 and 1B2). However, no statistically significant nonlinear patterns were observed (all *P* for nonlinearity > 0.05).

**Fig. 2:**
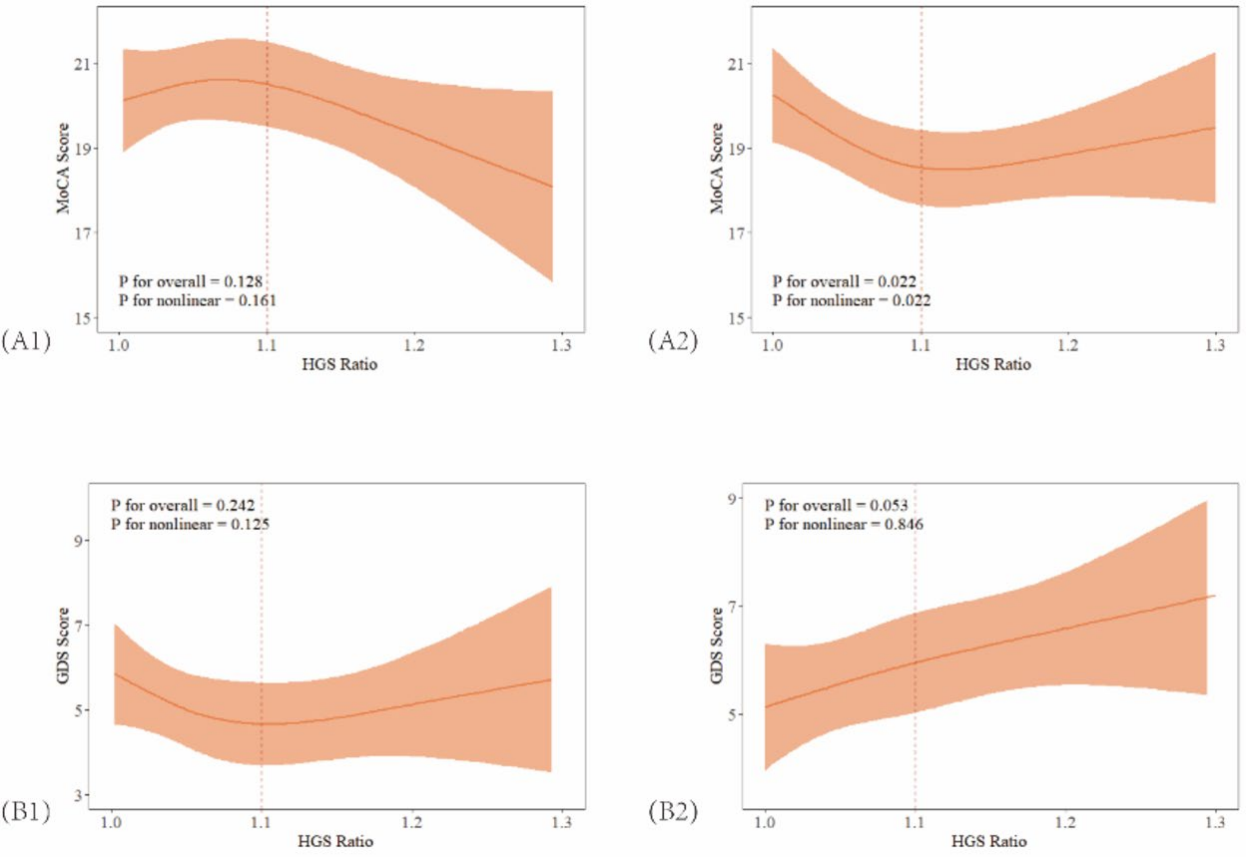
Associations of HGS Ratio with MoCA And GDS Scores. (A1) Association of HGS Ratio with MoCA Scores in Men. (A2) Association of HGS Ratio with MoCA Scores in Women. (B1) Association of HGS Ratio with GDS Scores in Men. (B2) Association of HGS Ratio with GDS Scores in Women. All adjusted for age, BMI, living arrangement, education levels, pre-retirement occupation, MNA scores, income. HGS ratio: handgrip strength ratio; MoCA: the Montreal Cognitive Assessment; GDS: the Geriatric Depression Scale; BMI: Body mass index; MNA: the Mini Nutritional Assessment.

We also analyzed the associations between the HGS ratio, MoCA scores, and GDS scores (Fig. 2). The RCS graph for men displayed a slight increase followed by a decrease in MoCA scores (P for nonlinearity = 0.161) (Fig. 2A1). In contrast, women demonstrated a significant L-shaped association, with the lowest MoCA scores at an HGS ratio of approximately 1.1 (P for nonlinearity = 0.022) (Fig. 2A2). Regarding GDS scores, men showed a trend where scores initially decreased and then increased, with the lowest point at an HGS ratio of approximately 1.1 (Fig. 2B1). Conversely, women displayed a positive association between the HGS ratio and GDS scores (Fig. 2B2). However, the nonlinear associations with GDS scores were not statistically significant for either sex (P for nonlinearity = 0.125 for men, P for nonlinearity = 0.846 for women).

In men, the prevalence of the four HGS status groups was 47.6% with neither weakness nor asymmetry, 25.4% with weakness alone, 15.2% with asymmetry alone, and 11.8% with weakness and asymmetry. The corresponding prevalence rates in women were 37.8%, 22.4%, 20.3%, and 19.4%, respectively. Participants in groups with HGS weakness alone, asymmetry alone, or weakness and asymmetry together, compared with those with neither weakness nor asymmetry, exhibited statistically significant characteristics in both sexes, including higher mean age, GDS, and MNA scores, lower dominant and non-dominant HGS values, and lower mean MoCA score (Table 2).

**Table 2:** Sex disparity in clinical and demographic characteristics with different HGS status groups.

	Men					Women				
Characteristic	Neither Weakness nor Asymmetry	Weakness Alone	Asymmetry Alone	Weakness and Asymmetry Together	*P*	Neither Weakness nor Asymmetry	Weakness Alone	Asymmetry Alone	Weakness and Asymmetry Together	*P*
n (%)	185 (47.6)	99 (25.4)	59 (15.2)	46 (11.8)		201 (37.8)	119 (22.4)	108 (20.3)	103 (19.4)	
Age(y)	70.92 ± 4.62	73.75 ± 5.53	71.05 ± 5.16	73.80 ± 5.50	< 0.001	70.24 ± 5.01	74.20 ± 6.34	70.82 ± 4.59	73.43 ± 6.25	< 0.001
BMI	23.25 ± 3.12	22.95 ± 3.95	23.80 ± 3.24	24.00 ± 3.38	0.238	24.14 ± 3.35	24.40 ± 3.90	23.64 ± 3.52	23.78 ± 3.43	0.343
MoCA	20.82 ± 4.47	18.93 ± 5.42	20.73 ± 4.45	17.17 ± 6.52	< 0.001	19.18 ± 5.60	15.51 ± 6.63	17.48 ± 5.74	14.57 ± 6.69	< 0.001
GDS	4,65 ± 4.11	6.46 ± 4.88	5.46 ± 3.83	5.09 ± 3.51	0.008	5.45 ± 4.57	6.30 ± 5.04	5.94 ± 4.57	7.38 ± 5.58	0.013
MNA	26.29 ± 2.05	25.47 ± 2.41	26.25 ± 2.04	26.19 ± 1.76	0.015	26.17 ± 2.06	25.42 ± 2.37	26.10 ± 1.88	25.35 ± 2.62	0.002
Dominant HGS	34.16 ± 3.96	23.49 ± 3.31	33.71 ± 3.78	22.24 ± 3.42	< 0.001	22.64 ± 3.36	13.79 ± 3.20	22.18 ± 2.96	13.65 ± 2.98	< 0.001
Non-dominant HGS	32.79 ± 3.91	22.60 ± 3.28	29.13 ± 3.74	19.15 ± 3.09	< 0.001	21.62 ± 3.25	13.17 ± 3.06	19.15 ± 2.83	11.75 ± 2.72	< 0.001

BMI: Body Mass Index; MoCA: the Montreal Cognitive Assessment; GDS: the Geriatric Depression Scale; MNA: the Mini Nutritional Assessment; Dominant HGS: dominant handgrip strength; Non-Dominant HGS: non-dominant handgrip strength.

After converting continuous MoCA and GDS scores into categorical cognitive impairment and depression variables, we conducted a multiple logistic regression analysis. The results revealed that HGS weakness alone, HGS asymmetry alone, and weakness and asymmetry together were all significantly associated with an increased risk of cognitive impairment in women (weakness alone: OR = 2.00, 95% CI = 1.17–3.37; asymmetry alone: OR = 1.93, 95% CI = 1.14–3.29; together: OR = 2.57, 95% CI = 1.48–4.46). However, no significant association was found between HGS and cognitive impairment in men. Additionally, no significant association was observed between HGS status and depression in the overall population of either sex (Table 3).

**Table 3:** Associations of HGS status with cognitive impairment, depression (reference = Neither weakness nor Asymmetry).

		Cognitive Impairment		Depression	
	n(%)	OR (95%CI)	*p*	OR (95%CI)	*p*
**Men (n = 389)**					
Neither Weakness nor Asymmetry	185 (47.6)	reference		reference	
Weakness Alone	99 (25.4)	1.66 (0.93,2.98)	0.087	1.87 (0.84,4.13)	0.124
Asymmetry Alone	59 (15.2)	0.89 (0.43,1.82)	0.747	0.85 (0.29,2.48)	0.770
Weakness and Asymmetry Together	46 (11.8)	2.10 (0.95,4.62)	0.065	0.25 (0.05,1.27)	0.095
**Women (n = 531)**					
Neither Weakness nor Asymmetry	201 (37.8)	reference		reference	
Weakness Alone	119 (22.4)	2.00 (1.17,3.37)	0.011	1.08 (0.52,2.26)	0.844
Asymmetry Alone	108 (20.3)	1.93 (1.14,3.29)	0.015	1.10 (0.51,2.38)	0.812
Weakness and Asymmetry Together	103 (19.4)	2.57 (1.48,4.46)	0.001	1.19 (0.56,2.55)	0.655

OR: odds ratio; CI: confidence interval.

All adjusted for age, BMI, living arrangement, education levels, pre-retirement occupation, MNA scores, income.

## Discussion

The correlation between HGS and the significant physiological and psychological changes in older adults is of growing interest to researchers. Our cross-sectional study indicates that higher dominant HGS and greater symmetry in HGS are linked to enhanced cognitive function and reduced depressive symptoms, particularly among women.

Our findings align with previous studies regarding the positive correlation between dominant HGS and MoCA scores^24–26^. Longitudinal studies have also revealed that individuals with weak HGS are at an increased risk of developing MCI or dementia^27–29^. Likewise, our results concerning the negative association between dominant HGS and GDS scores are consistent with other studies^30,31^. One potential mechanism for these associations could be regular physical activity, which not only maintains HGS but also stimulates the growth of prefrontal and hippocampal brain regions, potentially reducing cognitive decline and depression^32–36^. Another proposed explanation involves oxidative stress and inflammation, which are linked to cognitive performance, depression, and muscle mass, possibly mediating their association^37–41^. Our RCS models highlighted plateaus in the associations between dominant HGS and MoCA and GDS scores, indicating a threshold below which further decreases in dominant HGS significantly affect cognitive and depressive outcomes. This finding suggests that maintaining HGS above this threshold may be crucial for preventing cognitive decline and depression in older adults. These thresholds correspond to established cutoff points for HGS weakness, emphasizing the clinical relevance of these findings^18^.

Our analysis revealed that increased HGS asymmetry was associated with poorer cognitive performance and heightened depressive symptoms. While relevant studies are limited, some results support our findings^42,43^. One possible explanation is that HGS asymmetry may indicate an imbalance in hippocampal or frontal lobe volumes^44^or neural function^45^. The RCS models demonstrated associations between the HGS ratio, cognitive function, and depression. Critical inflection points differed between sexes, suggesting that women are more susceptible to the effects of HGS asymmetry. Additionally, women with HGS weakness alone, HGS asymmetry alone, or both exhibited a higher risk of cognitive impairment than those without these conditions, a pattern not observed in men. This effect is supported by a Japanese study that found a more pronounced link between skeletal muscle health and cognitive function in women^46^. Recent longitudinal studies have demonstrated that low HGS in women shows stronger predictive value for subsequent Activities of Daily Living (ADL) disability compared to men^47,48^. However, our study did not include an analysis related to ADL. The biological basis of these sex differences has not been fully elucidated but may involve factors such as the smaller gray matter volume in women^49^, making them more prone to rapid declines in gray matter volume associated with aging and uneven losses in brain regions linked to neurodegenerative diseases^50,51^. Additionally, sex hormones have been proposed as contributing factors^52^. These insights underscore the critical need to consider sex-specific risk factors when assessing and preventing cognitive decline and depression.

This study has several strengths. Firstly, it explored both separate and combined associations of HGS asymmetry and weakness with the risk of cognitive impairment and depression in older adults, providing comprehensive insights into these associations. Secondly, sex-stratified analyses were conducted to explore the potential effect of sex on the associations between HGS, cognition, and depression. Additionally, the utilization of standardized and widely recognized assessment tools, such as the MoCA and GDS, enhances the reliability and validity of the findings, facilitating direct comparisons with other studies. However, several limitations should be noted. Firstly, the cross-sectional design of the study limited the ability to infer causality between HGS status, cognitive decline, and depressive symptoms. Secondly, the generalizability of the study may be constrained by the restricted sample size and the exclusion of certain participants, particularly men. Thirdly, the absence of comprehensive ADL assessments precludes analysis of whether functional independence mediates the observed associations between HGS and cognitive function. Furthermore, information regarding hand dominance was absent in our study. Consequently, the hand with the highest HGS was designated as the dominant hand, potentially resulting in our handgrip ratio starting at 1.0 without variation below it, which differs from other studies where the handgrip ratio was distributed around 1.0, encompassing values both above and below. Nevertheless, a definition similar to ours has been used in some studies^53^. Additionally, there was a significant amount of missing data regarding comorbid conditions such as hypertension, stroke, and diabetes in our study, which limits our ability to conduct a more in-depth analysis^54^.

## Conclusion

These findings highlight the importance of jointly evaluating the strength and symmetry of HGS in relation to cognitive impairment and depression, particularly among women. Future investigations should delve deeper into the causal association between HGS status and adverse health outcomes.

## Data Availability

The datasets generated during and/or analysed during the current study available from the corresponding author on reasonable request.
